# Cannabinoid use and effects in patients with epidermolysis bullosa: an international cross-sectional survey study

**DOI:** 10.1186/s13023-021-02010-0

**Published:** 2021-09-06

**Authors:** Nicholas H. B. Schräder, Emily S. Gorell, Roy E. Stewart, José C. Duipmans, Nicole Harris, Victoria A. Perez, Jean Y. Tang, André P. Wolff, Marieke C. Bolling

**Affiliations:** 1 Department of Dermatology, University Medical Centre Groningen, University of Groningen, Groningen, The Netherlands; 2 Department of Dermatology, University of Cincinnati College of Medicine, Cincinnati, OH USA; 3 Department of Dermatology, Stanford University School of Medicine, Redwood City, CA USA; 4 Department of Health Sciences, Community and Occupational Medicine, University of Groningen, University Medical Centre Groningen, Groningen, The Netherlands; 5 Columbia University School of Medicine, New York, NY USA; 6 Anaesthesiology Pain Centre, Department of Anaesthesiology, University Medical Centre Groningen, University of Groningen, Groningen, The Netherlands

**Keywords:** Epidermolysis bullosa, Cannabinoid-based medicines, Symptom alleviation, Patient driven research, Genodermatoses, Survey, Pain, Itch, Wounds

## Abstract

**Background:**

Epidermolysis bullosa (EB) patient anecdotes and case reports indicate that cannabinoid-based medicines (CBMs) may alleviate pain and pruritus and improve wound healing. CBM use has not been characterized in the EB patient population.

**Objectives:**

To evaluate CBM use among EB patients, including CBM types, effects on symptoms (e.g., pain and pruritus), disease process (e.g., blistering, wounds, and inflammation), well-being (e.g., sleep, appetite) and concomitant medications.

**Methods:**

English-speaking EB patients or caregivers completed an online international, anonymous, cross-sectional survey regarding CBM use. Respondents reported the types of CBMs, subsequent effects including perceived EB symptom alteration, changes in medication use, and side effects.

**Results:**

Seventy-one EB patients from five continents reported using or having used CBMs to treat their EB. Missing question responses ranged between 0 (0%) and 33 (46%). Most used more than one CBM preparation (mean: 2.4 ± 1.5) and route of administration (mean: 2.1 ± 1.1). Topical and ingested were the most common routes. Pain and pruritus were reported retrospectively to decrease by 3 points (scale: 0–10; p < 0.001 for both) after CBM use. Most reported that CBM use improved their *overall EB symptoms* (95%), *pain* (94%), *pruritus* (91%) and *wound healing* (81%). Most participants (79%) reported decreased use of pain medications. The most common side-effect was dry mouth (44%).

**Conclusions:**

CBMs improve the perception of pain, pruritus, wound healing, and well-being in EB patients and reduced concomitant medication use. Nevertheless, a direct relation between the use of CBMs and reduction of the above-mentioned symptoms cannot be proven by these data. Therefore, future controlled studies using pharmaceutically standardised CBM preparations in EB are warranted to delineate the risks and benefits of CBMs.

**Supplementary Information:**

The online version contains supplementary material available at 10.1186/s13023-021-02010-0.

## Background

Epidermolysis bullosa (EB) is a group of clinically and genetically heterogeneous genetic skin conditions whereby patients have fragile skin and mucosae that blister with slight trauma [1]. EB is characterized by repeated blistering and wounding, often with impaired wound healing, leading to a cascade of secondary problems, including chronic wounds, scarring, deformities, infections and extracutaneous symptoms [2]. EB is divided into types according to the level of blister formation, including EB simplex (EBS), junctional EB (JEB) and dystrophic EB (DEB), which is further subdivided into recessive DEB (RDEB) and dominant DEB (DDEB) [1].

Pain and pruritus have a significant impact on quality of life in EB patients; two of the top three unmet needs for EB patients include effective pain and pruritus treatments [3–6]. EB patients and clinicians continually seek out novel treatments to improve symptomatic care and quality of life. Two recent case series have brought to light the use of cannabinoid-based medicines (CBMs) in the EB-care setting [7–9]. However, the therapeutic potential and risks of such drugs have yet to be delineated in the context of EB care.

Cannabinoids, whether endogenous (endocannabinoids), plant-derived (phytocannabinoids), or synthetic, are molecules that activate various cascading pathways through endogenous cannabinoid-binding receptors (CB1 and CB2) and transient receptor potential (TRP) ion channels within the endocannabinoid system (ECS) [10]. These receptors are ubiquitous in the central and peripheral nervous systems, but are also localized to the immune system and skin [10–12]. Over 100 cannabinoids have been isolated, however Δ9-tetrahydrocannabinol (THC) and cannabidiol (CBD) are the most abundant and well-studied active cannabinoids [13]. THC, known for its psychoactive effects, is a partial agonist of CB1 and CB2. CBD, which is psychotropically inert, antagonizes THC effects, improving the tolerability of THC when co-administered [14].

Pain treatment with CBMs has been well-described. Literature reviews of clinical studies have shown CBMs to be tolerable, reasonable therapeutic options for chronic pain in various conditions [15–17]. On the contrary, the effects of CBMs on pruritus lack sufficient clinical studies, but underlying mechanisms are attributed to neuronal activation, and local effects on keratinocytes and mast cells [18–22]. The modulation of pain and pruritus through CB1/CB2 ligation warrants further research on their potential role in alleviating symptoms and has the potential to become a novel target for treatment in EB [9, 23].

To improve our understanding of CBM treatments currently being used in EB, and give direction to future research, we report the results of a survey aimed at highlighting patient-reported CBM-use, and self-reported effects on their EB.

## Patients and methods

Eligibility included all types of inherited EB, all ages, and any geographic location. The survey was available online in English. Caregivers or parents could complete the survey on a patient’s behalf. Participants 18 years old or older provided electronic informed consent. Assent and parental informed consent were completed for respondents aged 7–18 years. Parental informed consent was obtained for participants less than 7 years old.

The survey link was shared among EB-related social media groups and EB non-profit organization web pages and newsletters. Survey information was distributed among professional networks (EB Clinical Research Consortium and EB Clinical Network) who shared the information with EB patients.

The cross-sectional online survey obtained self-reported data on demographics, disease characteristics, CBM characteristics, effects of CBMs on EB symptoms, side effects, and changes in concomitant medication use (Additional file 1: Appendix 1). Survey questions were developed based on previously reported effects of CBMs on EB and other conditions, as well as input from EB patients and expert physicians [7, 8]. As this was an internationally disseminated survey, given the potential illegality of CBMs, it was designed to uphold participant anonymity. The survey utilized skip logic whereby pertinent follow up responses could be elicited. Retrospective pain and pruritus levels pre- and post-CBM use were collected as numerical rating scales (NRS), ranging from 0 to 10 [24]. The Wong-Baker FACES scale was included to assist with self-reporting of pain [25]. A 5-point Likert scale was used to assess improvement or worsening of EB symptoms; a 6-point Likert scale was used to assess medication changes. Options for “Not Applicable” were included for EB symptoms and medication use. An option to upload the CBM product label was included to confirm reported content of the product(s) used. Respondents had the option to include free text comments, which were reviewed by study staff. Qualitative answers were assessed for predominant themes.

Survey responses were collected, and data was managed using REDCap (Research Electronic Data Capture) secure electronic data capture tools hosted at Stanford University. Both the study and survey instrument were approved by the Stanford Institutional Review Board (Protocol #53145) and the Groningen Institutional Ethics Review Board. Survey responses were collected from March through August 2020.

To ensure that only unique survey completions were included, surveys with identical responses for demographics or qualitative responses were identified and reviewed; the survey with the fewest completed questions was removed. Each individual survey was reviewed to confirm that responses appeared credible. Survey respondents that reported no previous or current CBM use were excluded. Incomplete surveys were included, however those that failed to complete at least the sections on CBM characteristics were excluded.

Levels of pain and pruritus pre- and post-CBM use were compared by utilizing non-parametric tests. Non-parametric testing was also applied to group comparisons. For costs, class midpoints from ordinal responses were extracted, and foreign currencies converted to United States Dollars based on exchange rates in December 2020. Statistical analyses were performed using SPSS (version 23.0).

## Results

### Demographics

In total, 155 surveys were returned. Of these, 84 surveys were excluded based on either incompletion (n = 42), no CBM use reported (n = 40), or were identified as duplicates (n = 2). The remaining 71 (45.8%) survey responses comprised the analyzed study cohort (Table 1). Surveys were completed more frequently by EB patients (43/71, 60.6%) than parents/caregivers (28/71, 39.4%). Most responses came from North America (62/71, 87.3%) but the cohort included inhabitants of five continents. The majority were ≥ 18 years old (45/71, 63.4%). Participants with RDEB (37/51, 52.1%) comprised the largest represented EB type. Most participants reported *moderate* to *very severe* EB severity (58/71, 81.7%). The vast majority of participants (62/71, 87.3%) currently used CBMs, versus those that had stopped using CBMs. Reasons for discontinuation included: CBMs were too expensive (n = 4), a better medication was found (n = 3), and that CBMs were illegal (n = 1). The median monthly costs for CBMs were USD $75.00 (IQR $66.84-$150).

**Table 1:** Demographics and characteristics of survey respondents

	Total cohort	RDEB	DDEB	EBS	JEB
**Participants enrolled, n (%)**	71 (100.0)	37 (52.1)	8 (11.3)	17 (23.9)	8 (11.3)
**Participant role, n (%)**					
**Patient**	43 (60.6)	18 (48.6)	6 (75.0)	13 (76.5)	5 (62.5)
Parent/guardian/caregiver	28 (39.4)	19 (51.4)	2 (25.0)	4 (23.5)	3 (37.5)
**Age, n (%)**					
< 7	11 (15.5)	5 (13.5)	1 (12.5)	3 (17.6)	2 (25.0)
7–12	8 (11.3)	7 (18.9)	–	1 (5.9)	–
13–17	7 (9.9)	7 (18.9)	–	–	–
18–25	10 (14.1)	7 (18.9)	3 (37.5)	–	–
26–34	14 (19.7)	6 (16.2)	1 (12.5)	5 (29.4)	2 (25.0)
> 34	21 (29.6)	5 (13.5)	3 (37.5)	8 (47.1)	4 (50.0)
**Sex, n (%)**					
Male	40 (56.3)	25 (67.6)	4 (50.0)	7 (41.2)	3 (37.5)
Female	31 (43.7)	12 (32.4)	4 (50.0)	10 (58.8)	5 (62.5)
**Geographic region, n (%)**					
North America	62 (87.3)	33 (89.2)	7 (87.5)	16 (94.1)	5 (62.5)
Europe	5 (7.0)	2 (5.4)	1 (12.5)	1 (5.9)	1 (12.5)
Oceania	2 (2.8)	1 (2.7)	–	–	1 (12.5)
Asia	1 (1.4)	1 (2.7)	–	–	–
Africa	1 (1.4)	–	–	–	1 (12.5)
**Self-reported severity, n (%)**					
Very mild	1 (1.4)	–	–	1 (5.9)	–
Mild	12 (16.9)	2 (5.4)	3 (37.5)	7 (41.2)	–
Moderate	22 (31.0)	9 (24.3)	3 (37.5)	6 (35.3)	3 (37.5)
Severe	24 (33.8)	18 (48.6)	1 (12.5)	3 (17.6)	2 (25.0)
Very severe	12 (16.9)	8 (21.6)	1 (12.5)	–	3 (37.5)
**CBM-use, n (%)**					
Currently administering CBM	62 (87.3)	33 (89.2)	6 (75.0)	14 (82.4)	8 (100.0)
Previously administered CBM	9 (12.7)	4 (10.8)	2 (25.0)	3 (17.6)	–
**Age when CBM started, median (IQR)**	19 (14.0–26.0)	16 (11.8–24.3)	18 (10.0–21.5)	25 (12.5–38.0)	25.5 (21.8–44.5)
**Monthly costs in USD, median (IQR) (n = 49)**	$75.00 ($66.84–$150)	$117.33 ($75.00–$150.00)	$112.50 ($33.42–$150.00)	$75.00 ($27.15–$150.00)	$75.00 ($25.00–$91.93)

Demographics, participant reported EB characteristics and CBM-use status of survey respondents (n = 71), grouped by EB types. One participant with unknown EB type was removed from EB type columns

RDEB: recessive dystrophic epidermolysis bullosa, DDEB: dominant dystrophic epidermolysis bullosa, EBS: epidermolysis bullosa simplex, JEB: junctional epidermolysis bullosa, CBM: cannabinoid-based medicine, IQR: inter-quartile range, USD: United States dollar

### Cannabinoid-based medicine characteristics

Participants reported characteristics of the CBMs administered and were able to report on multiple CBMs (Table 2). *Oil/paste, flos* and *edible* (infused or cooked into foods) were the most prevalent CBM preparations. On average, individual participants administered 2.4 (± 1.5) CBM preparation types. *Topical* (36/60, 60.0%) and *ingested* (36/60, 60.0%) were the most frequently encountered routes of administration. On average, participants used 2.1 (± 1.1) administration routes. More respondents (18/44, 40.9%) preferred the *inhaled* route compared to other routes. For participants under 13 years, topical administration was most frequently used (12/19, 63.2%).

**Table 2:** Characteristics of cannabinoid-based medicines

Survey item subject (number of responses to item)	Multiple choice options for survey responses	All respondents (%)	Respondents < 13 years (%)
CBM type* N all respondents = 60 N < 13y = 19	Oil/paste	46 (76.7)	11 (57.9)
	Dried flower/Flos	33 (55.0)	2 (10.5)
	Edible	29 (48.3)	2 (10.5)
	Tincture	17 (28.3)	3 (15.8)
	Other liquid form	11 (18.3)	1 (5.3)
	Pill	5 (8.3)	–
	Total CBM types used	141	19
	Mean per participant	2.4 (± 1.5)	1.0 (± 0.7)
CBM route of administration* N all respondents = 60 N < 13y = 19	Topical	36 (60.0)	12 (63.2)
	Ingested	36 (60.0)	4 (21.1)
	Inhaled	33 (55.0)	–
	Sublingual	23 (38.3)	4 (21.1)
	Total routes reported	128	20
	Mean per participant	2.1 (± 1.1)	1.1 (± 0.8)
Topical CBM type* N all respondents = 36 N < 13y = 19	Cream	24 (66.7)	8 (42.1)
	Oil	23 (63.9)	6 (31.6)
	Lotion	12 (33.3)	4 (21.1)
	Spray	3 (8.3)	1 (5.3)
	Foam	–	–
	Total types of topicals	62	19
	Mean per participant	1.7 (± 1.1)	1.1 (± 1.2)
Composition of CBM (THC vs. CBD)* N All Respondents = 58 N < 13y = 19	THC only	18 (15.3)	–
	THC/CBD combination	41 (34.7)	6 (31.6)
	CBD only	24 (20.3)	9 (47.4)
	Unknown	35 (29.7)	4 (21.1)
	Total compositions reported	118	19
Current frequency of CBM administration N all respondents = 51 N < 13y = 10	Less than once per week	5 (9.8)	2 (20.0)
	Once per week	2 (3.9)	–
	Several times per week	8 (15.7)	1 (10.0)
	Once per day	15 (29.4)	4 (40.0)
	Several times per day	19 (37.3)	2 (20.0)
	Unsure	2 (3.9)	1 (10.0)
Change over time: dose of CBM N all respondents = 52 N < 13y = 11	Dose decreased	6 (11.5)	1 (9.1)
	Dose remained stable	13 (25.0)	2 (18.2)
	Dose increased	13 (25.0)	4 (36.4)
	Dose fluctuated	17 (32.7)	2 (18.2)
	Unsure	3 (5.8)	2 (18.2)
Change over time: frequency of CBM administration N all respondents = 52 N < 13y = 11	Administered less frequently	7 (13.5)	1 (9.1)
	Frequency remained stable	17 (32.7)	3 (27.3)
	Administered more frequently	9 (17.3)	2 (18.2)
	Administration frequency fluctuated	17 (32.7)	4 (36.4)
	Unsure	2 (3.8)	1
CBM source of acquisition* N All Respondents = 50 N < 13y = 10	Cannabis dispensary	31 (62.0)	6 (60.0)
	Social connection	19 (38.0)	2 (20.0)
	Internet web-shops	6 (12.0)	4 (40.0)
	Medical pharmacy	5 (10.0)	1 (10.0)
	Cultivated at home	4 (8.0)	–
	Prefer not to answer	1 (2.0)	–
	Total number of sources	66 (100)	13 (100)
Preferred route of administration N all respondents = 44 N < 13y = 9	Topical	11 (25.0)	4 (44.4)
	Inhaled	18 (40.9)	
	Ingested	10 (22.7)	2 (22.2)
	Sublingual	5 (11.4)	3 (33.3)
Duration of use N all respondents = 52 N < 13y = 11	< 6 months 6 months–1 year 1–5 years > 5 years	13 (25.0) 7 (13.5) 18 (34.6) 14 (26.9)	5 (45.5) 4 (36.4) 2 (18.2) –
Reported side effects* N all respondents = 45 N < 13y = 9	Dry mouth	20 (44.4)	–
	Cough/wheezing	13 (28.8)	–
	Dry/red eyes	12 (26.7)	1 (11.1)
	Fatigue	10 (22.2)	–
	Dizziness	7 (15.6)	–
	Paranoia	3 (6.7)	–
	Problems with memory/attention	3 (6.7)	–
	Problems with coordination	3 (6.7)	–
CBM prescribed by physician N all respondents = 50 N < 13y = 10	Yes	12 (24.0)	1 (10.0)
	No	38 (76.0)	9 (90.0)
Physician aware of CBM use N all respondents = 50 N < 13y = 9	Yes	28 (73.7)	7 (77.8)
	No	10 (26.3)	2 (22.2)

Reported cannabinoid-based medicines (CBMs) and characteristics of administration in the total cohort. * Indicates that participants were able to select more than one response. Participants were not required to respond to each item. Administration by way of suppositories were not reported by any participant

THC: delta-9-tetrahydrocannabinol, CBD: cannabidiol

Most commonly, CBM products contained both THC and CBD (41/118, 34.7%), followed by CBD-only (24/118, 20.3%) and THC-only (18/118, 15.3%). An unknown cannabinoid content was reported for 29.7% of CBMs (35/118). The largest group within respondents under 13 years used CBD-only (9/19, 47.4%).

The majority of participants (34/51, 66.7%) administered CBMs at least once daily. The largest group reported that their dose of CBM fluctuated over time (17/52, 32.7%), followed by both stable and increased (13/52, 25.0%), and decreased (6/52, 11.5%). For frequencies of CBM usage, most participants reported either *stable* or *fluctuating* (17/52, 32.7%) frequencies.

CBMs were acquired primarily through dispensaries (31/50, 47.0%) and social connections (19/50, 28.8%). A minority of CBMs were prescribed (12/50, 24.0%), however most participants (28/50, 73.7%) reported that their clinicians were aware of their CBM use. Within the group of 12 participants using prescribed CBMs, 9 were from the United States, 2 from the Netherlands, and 1 from Australia.

Reasons for initiating CBM use were reported by 63 patients as a qualitative open-ended response. Of these responses, the most cited reason to start using a CBM was to treat pain (n = 40), and other EB symptoms, such as wound healing (n = 11), to improve sleep (n = 5), and to treat pruritus (n = 5). Additionally, respondents reported starting CBM as an alternative to opiates (n = 8), inadequate control with non-CBM regimen (n = 7), and intolerable side effects for non-CBM regimen (n = 6).

### Reported effects of cannabinoid-based medicines

**Fig. 1:**
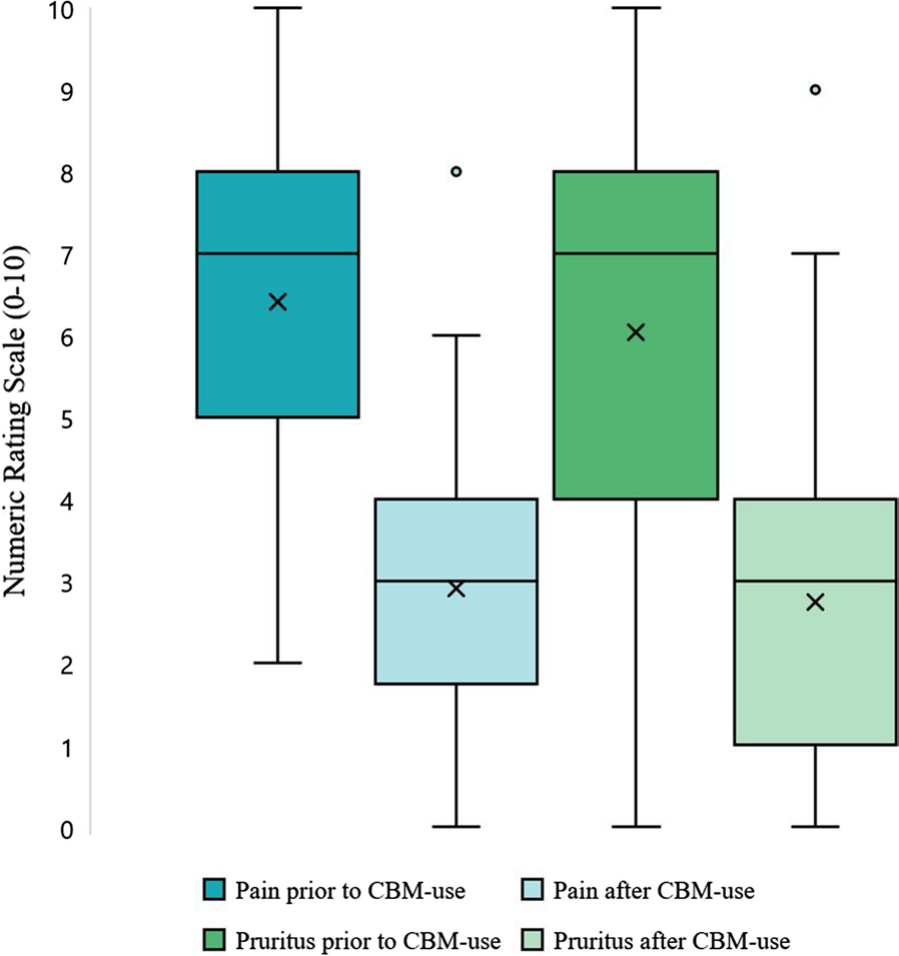
Boxplot of numeric rating scale (NRS 0–10) scores for pain and pruritus prior to- and after cannabinoid-based medication (CBM) administration. Note the improvement in the perception of both pain and pruritus following CBM use. Significant differences (p < 0.001) were seen for changes in pain and pruritus (*prior to CBM-use* vs. *after CBM-use*)

Statistically significant reductions in self-reported pain and pruritus were reported retrospectively following CBM use (median pain change-score: − 3, IQR: − 2 to − 4 [p < 0.001, 95% CI 2.95–4.04], median pruritus change-score: − 3, IQR: − 1.25 to − 5 [p < 0.001, 95% CI 2.59–3.10]) (Fig. 1). There were no significant differences in the change scores for pain and pruritus between EB types (pain: p = 0.837, pruritus: p = 0.864) or self-reported disease severity (pain: p = 0.644, pruritus: p = 0.962). As most participants reported multiple CBM routes of administration and formulations, it was not possible to analyse degrees of improvement based on these variables.

**Fig. 2:**
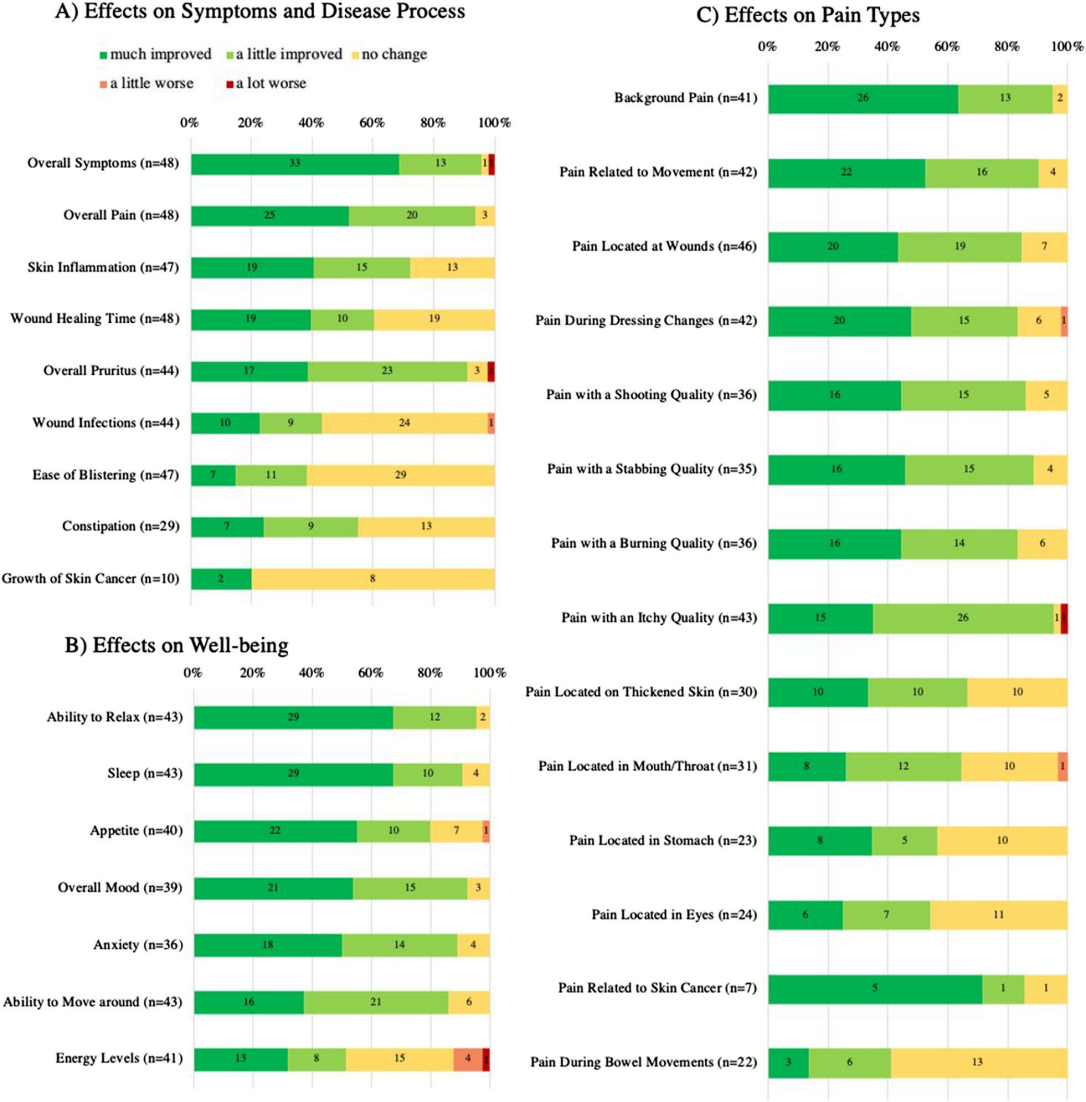
Participant-reported effects of cannabinoid-based medicines on epidermolysis bullosa-related **A** symptoms and disease process, **B** well-being and **C** pain types. Data is ordered by frequency of *much improved*. N = 48 responses are included. Items indicated as *not applicable* by participants are removed

The vast majority of participants reported improvement (*much improved* or *a little improved*) in *overall symptoms* (46/48, 95.8%), *overall pain* (45/48, 93.8%) and *overall pruritus* (40/44, 90.9%) from CBM administration (Fig. 2)*.* To a lesser extent, *skin inflammation* (34/47, 72.3%) and *wound healing time* (29/48, 60.4%) were reportedly improved with CBMs.

Within effects specific to pain types and qualities, most participants reported improvements in *background pain* (39/41, 95.1%)*, pain related to movement* (38/42, 90.5%), *pain located at wounds* (39/46, 84.8%), *pain during dressing changes* (35/42, 83.3%) as well as *itchy* (41/43, 95.3%)*, shooting* (31/36, 86.1%)*, stabbing* (31/35, 88.6%) and *burning* (30/36, 83.3%) qualities of pain.

The *ability to relax* (41/43, 95.3%), *overall mood* (36/39, 92.3%), *improvement in anxiety* (32/36, 88.9%), *sleep* (39/43, 90.7%), and *ability to move around* (37/43, 86%) were the most frequently improved effects related to participants’ well-being.

Of participants who reported having skin cancer (n = 10), two indicated reduced cancer growth. Of the seven who reported experiencing pain from skin cancer, six (85.7%) denoted decreased pain from skin cancer with CBM use.

Notably, a small fraction of responses indicated worsening in *overall symptoms* (1/48, 2%), *overall pruritus* (1/44, 2.3%), *wound infections* (1/44, 2.3%), *pain during dressing changes* (1/42, 2.4%), *pain with an itchy quality* (1/43, 2.3%), *pain in the mouth/throat* (1/31, 3.2%), *appetite* (1/40, 2.5%), and *energy levels* (5/41, 12.2%) (Fig. 2)*.*

**Fig. 3:**
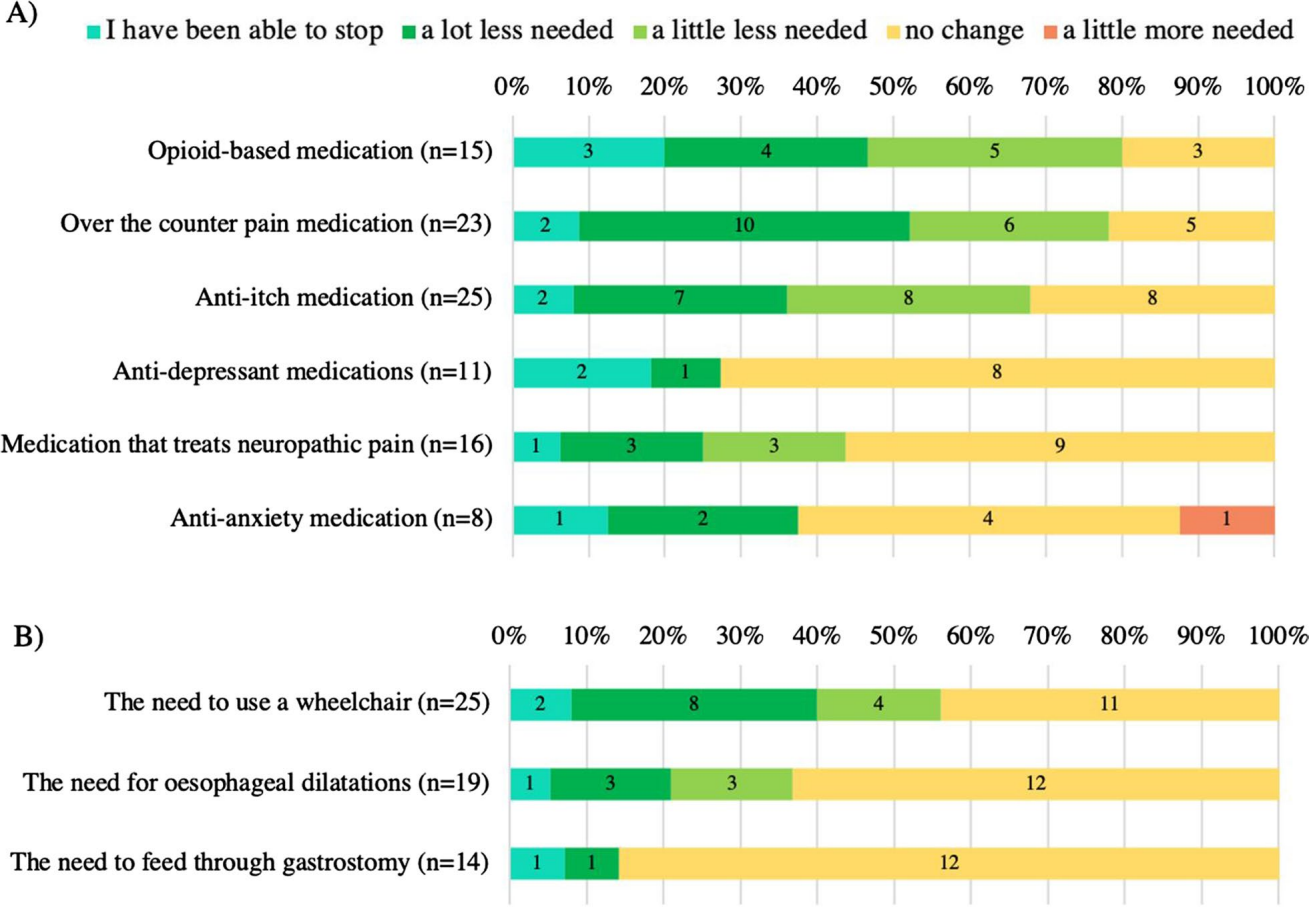
**A** Reported changes in pain and pruritus treatments due to cannabinoid-based medication administration. **B** Reported changes in supportive interventions. Note that not all participants answered each question. No participants indicated “A lot more needed” for any responses

Effects of CBM administration on pain and pruritus treatments were highlighted by cessation of, or reduction in the use of, opioids (12/15, 80.0%), over the counter pain medications (18/23, 78.3%) and anti-itch medications (17/25, 68.0%, Fig. 3). Of participants who required wheelchair assistance, most (14/25, 56.0%) reported a reduced need to use a wheelchair.

The most commonly encountered side-effect from CBM administration was *dry mouth* reported by 44% of participants (Table 2). *Cough/wheezing* (29%), and *dry/red eyes* (27%) were the next most prevalent side effects.

## Discussion

We report the first international summary of patient reported CBM use in EB. The literature assessing CBM treatments in EB is non-existent. To date only two small (n = 6 total) retrospective case-series have been published, which highlighted various cannabinoid compositions of CBMs, administered topically or sublingually [7, 8]. The reported effects here were strikingly similar, characterized by reductions in pain, pruritus, and in the use of other systemic medications, such as opioids. In EB, pain treatment guidelines have noted the use of CBMs as potential modalities, but evidence remains empirical [4].

EB is an immensely burdensome condition from birth, which, in the absence of a cure or highly effective treatments, means that patients are on a continuous journey to find relief in novel, repurposed or alternative treatments [26]. It is not surprising that EB patients willingly experiment with treatments that may not have been thoroughly investigated, such as CBMs that contain CBD and THC. Several participants reportedly sought out CBMs as an alternative to conventional medications (such as opioids), or other substances (such as alcohol). Reassuringly the majority informed their clinicians of their CBM use. However, even with promising anecdotes, before any clinical decisions can be made regarding their safety, tolerability and effectiveness, CBM treatment risks and benefits should be clearly delineated.

Most of the responses in this study were from North America, despite international outreach to recruit respondents; four other continents were also represented. Due to the extensive availability of CBMs in the United States and Canada, it was fitting that the majority of responses originated from these two countries [27, 28].

The vast majority of respondents had RDEB, and half of participants reported their EB as *severe* or *very severe*. Although each EB type has a unique pathophysiology, some symptoms, such as blistering and wound formation, overlap. Patients with any EB type can perceive their disease as severe, influenced by environmental and psychosocial factors; it appears that this perception of disease severity likely contributed to participant willingness to try CBMs [29]. However, reported improvement in symptomatology was not exclusive to RDEB, nor was it seen only in patients who self-classified as *severe*.

The most commonly cited reason for discontinuing CBMs was price, with an average of USD $75 spent monthly. A downside to the burgeoning market of CBM products is the expense, especially in a population already significantly impacted by the financial burden of caring for their disease [30, 31]. Notably, some participants in the United States reported that they started using CBMs due to difficulties in obtaining conventional allopathic medications, either due to price, lack of insurance coverage, or difficulty obtaining prescriptions (often narcotics).

### Characteristics of cannabinoid-based medicines

The administration routes reported in this study were predominantly inhaled, topical and oral, closely followed by sublingual. Generally, inhaled and sublingual CBM administration lead to the fastest plasma cannabinoid peaks, due to the direct absorption of cannabinoids into the vasculature, allowing for efficient dose-titration in shorter time-frames [32, 33]. This may partially explain why participants in this study mostly preferred the inhaled route.

Topical CBMs, however, have local effects, whereby psychotropic or systemic side-effects are very limited, but the mechanisms of peripheral CB1/2 binding may differ from systemic CBMs [34]. Patients or caregivers may initiate these CBM treatments due to the ease of topical administration, the high burden of skin related problems in EB, and the desire to minimize side effects, as was reported in a case study [7]. We found that topical administration of CBD-only products were the predominant CBMs used for children with EB. The increased availability of legal CBD-only products may have also contributed to the increased use of these products compared to other CBMs.

Most participants administered CBMs at least daily, underscoring the degree of intervention required to manage EB symptoms. The reported dosages and frequency of use showed heterogeneity in change over time. A small, yet significant number of participants noted an increase in both administered dose (13/52, 25%) and administration frequency (9/52, 17.3%) over time. Typically, tolerance to the efficacy of CBMs containing THC and CBD, has not been observed [35–38]. However, tolerance is developed to known effects from cannabinoid receptor agonism such as impaired neurocognition and cardiovascular changes [39]. The changes in dose and frequency of administration seen in this study may also be due to the dynamic natural history of symptoms in EB, such as the development or resolution of blistering and chronic or recurrent wounds, fluctuations in pain and pruritus, or disease progression, amongst an array of factors [2, 40].

There is ongoing debate as to the synergistic value of combining multiple cannabinoids (i.e., both CBD and THC) in CBM treatments [41]. The phytocannabinoids THC and CBD, amongst other cannabinoids, have unique binding properties with CB1/2 and other ECS-associated receptors. THC is proposed as the molecule central to CBM pain treatments, however current recommendations include adding CBD to mitigate THC-mediated side effects [42]. Increasing ratios of THC:CBD linearly improve patient-reported effects for numerous indications, but could reduce these effects at too high a ratio [43]. The varying cannabinoid compositions used by participants in our study may be due to differences in legality, availability, recommendations from peers, or settings where participants acquired CBMs. Standardizing CBM compositions, as well as finding the optimal ratios for such treatments will help gain more accurate insights into CBM effects in EB.

### Effects on symptoms of epidermolysis bullosa

Both the highest prevalence and proportion of reported improvement from CBMs was in *overall symptoms*. This was closely followed by *overall pain* and *pruritus*. As participants indicated multidimensional positive effects of CBMs in this study, it is likely that the synergistic improvements in multiple aspects of their disease may together be more clinically meaningful than each individual effect. Contrastingly, very few patients reported worsening of symptoms.

The greatest degrees of improvement were observed in *background pain*, *pain related to movement* and *wound pain*. Some of the most well studied indications for CBM interventions are chronic pain conditions [17, 44]. The consistently superior results of CBMs, containing both THC and CBD, versus placebo in non-EB neuropathic pain studies is promising, but is overshadowed by lack of high-quality evidence [45]. To date, CBMs have, at most, moderate quality evidence supporting their effectiveness for chronic pain. Pain, however, is a complex experience, influenced by neurobiological and psychosocial mechanisms [46]. The aetiology of pain in EB can be multifactorial including nociceptive pain (e.g., acute wounds, chronic wounds, dressing changes, surgical interventions, and extra-cutaneous sites of tissue damage), continuous background pain, neuropathic and nociplastic pain [4]. Due to the heterogeneity of pain mechanisms and responses to CBMs, establishing therapeutic mechanisms of CBMs in EB will be a challenging feat, but one deserving of additional study.

Many respondents reported overall reductions in pruritus. Patient-centred research has shown that pruritus is one of the most bothersome symptoms in EB [6, 47, 48]. Despite numerous treatment modalities, alleviation is challenging [5, 49]. Thus, pruritus relief by way of intervention with CBMs could be especially meaningful to patients. Decreased pruritus was also noted in the previous case report of sublingual CBM use in EB patients [8]. However, one patient in our study did note increased pruritus as a side effect of CBMs. CBMs have reportedly decreased pruritus in multiple conditions such as atopic dermatitis, psoriasis, prurigo nodularis, uremic pruritus, and lichen amyloidosis [20, 50]. It is postulated that the anti-pruritic actions of CBMs, like in pain, are due to effects within the ECS including ligation of CB1/2 receptors, and TRP channel modulation [19, 20, 22]. Additional studies are warranted to investigate pruritus alterations with CBMs in EB, including elucidating the most effective routes of administration, and CBM compositions.

The wound environment in EB consists of an interplay between intrinsically impaired wound healing, bacterial colonization, inflammation and the external wound environment [51]. The majority of participants reported improved wound healing and inflammation from CBMs. After tissue injury, the ECS plays an intricate role in the regulation of cytokines, nitrergic signalling and keratinocyte differentiation, through direct and indirect activation of cannabinoid receptor ligands [22, 52–54]. The two recent case series, coupled with our results deem further investigation necessary to address whether CBM modulation of the ECS is beneficial for EB wounds [7, 8]. We do note that subjectively reported improvements in wound healing and inflammation could also be multifactorial, secondary to reductions in pain and/or pruritus. The natural history of EB wounds is often unpredictable and influenced by both biological and environmental factors [40, 55]. Future studies assessing the effects of CBMs on EB wounds should incorporate objective wound assessments.

The highest proportions of reductions in concomitant medications were seen in opioid-based analgesics, over the counter analgesics, and anti-itch medications. These treatments comprise one aspect of the multidimensional approach to symptomatic care in EB [4, 5]. Yet, often in EB, conventional medications do not provide adequate effectiveness and are eclipsed by their burdensome short and long term side-effect profiles [4, 56]. Identifying effective pain and pruritus treatments remains a research priority in EB [29, 57–59]. Recent reviews suggest a prominent opioid-sparing effect from CBMs, warranting future clinical studies to investigate a causal relationship [60]. Although CBMs do not appear to alter the human pharmacokinetics of opioids, the ECS and endogenous opioid systems share neuroanatomical, neurochemical and pharmacological characteristics [61, 62]. The reduction of concomitant analgesic and anti-pruritic medication in this study seems promising, and mirror those effects previously reported wherein some patients were able to discontinue opioids [7, 8]. However, it is of significant importance to obtain a clear picture of this phenomenon in EB by clarifying both the short and long-term risks and benefits of these medication regimen alterations.

Notably, of the respondents who required a wheelchair, more than half were able to decrease or even stop using their wheelchair. These respondents reported both improved *energy levels* and *ability to move around*. It is likely that these improvement are related to the reduction in symptomatic burdens, yet remains an important finding as increased mobility in EB has myriad conceivable benefits [63–65].

Ten participants reported skin cancer, of which two noted a reduction in growth with CBMs. There have been reports of anti-neoplastic effects of CBMs [12, 19, 66]. Unfortunately, we are not able to validate these findings, nor can we describe potential anti- or pro-neoplastic effects of CBMs, as no conclusive evidence has delineated the mechanisms of action, risks and benefits of such therapies in EB. Additionally, 6/7 participants reported reduced pain from skin cancer (the type of cancer was not specified), due to CBMs. Current literature suggests a role for CBMs in alleviating cancer-related pain in non-EB patients, however high quality evidence is lacking [67].

A large proportion of participants indicated a positive impact of CBMs on their well-being, of which the greatest reported improvements were for the *ability to relax* and *sleep*. Daily well-being has been given substantial consideration in EB best practice guidelines, and highlight the multidimensional burdens of living with EB [4, 68]. Whether these reported improvements are direct physiological effects of CBMs, or indirect through symptom alleviation, are not known and will require further investigation.

### Side effects

In this study, dry mouth was the most commonly reported side-effect, likely due to inhibition of salivation through CB1/2 modulation in the salivary glands, via THC and CBD-induced agonism [69]. Dry eyes were also reported, probably through similar mechanisms in lacrimal glands. Ocular, oral, and dental sequelae of EB can be exacerbated by these effects, and thus should be considered prior to CBM administration [70]. However, overall, scientific literature points out that CBMs are generally well tolerated, as was reported by participants in this study [71].

An additional factor to consider, with specific regard to THC, is CBM dependence and withdrawal symptoms after therapy cessation, or periods of cessation, which were not incorporated in this study. The lifetime risk for dependence after cannabis use in recreational settings (high THC concentrations and aim to achieve psychotropic effects) is 8.9% [72]. The dependence risk whilst using CBMs for therapeutic goals is however unknown. Withdrawal symptoms may include irritability, anger, anxiety, sleep difficulty, decreased appetite and weight loss, and is dose-dependent [73]. Future prospective controlled studies taking withdrawal into account, may add value to the sparse evidence on withdrawal after clinically supervised CBM-use.

### Limitations

Given the potential illegality of CBM use, we implemented an anonymous online survey. Thus, we were unable to gauge the prevalence of CBM use, nor a response rate. During the data cleaning phase, the research team cross-checked individual responses, but could not completely validate the content of submitted surveys.

The number of included patients was small for English speaking countries outside North America and may be due to differences in availability of CBMs. Unfortunately, we must also accept that CBM use, even with clinical supervision, carries a stigma whereby patients may refrain from disclosing their use.

The composition of the CBM products used by patients is also not entirely clear. While we attempted to ascertain the cannabinoid compositions of CBMs by allowing participants to upload product labels, not every participant uploaded their label. Additionally, the commercial market regarding CBMs remains unregulated whereby cannabinoid compositions are non-standardised and may be inaccurately labelled [74, 75]. In order to avoid health risks of accidentally administering unwanted additives, it is important for patients to acquire CBMs from reputable sources. This also underscores the potential benefit of regulation which would come with legalization of CBM products.

Furthermore, we unfortunately were unable to determine how cannabinoid concentrations (CBD versus THC) and route of administration contributed to the perceived efficacy of CBMs reported in this study, as most participants used multiple products and routes of administration. Additional areas of future exploration include those that were alluded to by free text comments. Various participants reported that certain formulations and routes may be more efficacious for different symptoms and scenarios: “We find that a combination of ingested and topical applications works best, as each product helps with a different aspect of EB. Ingested products help with internal inflammation and pain control. Topical products help with localized pain and wound healing.” Certainly, additional controlled studies are warranted to explore the complex relationships between cannabinoid compositions, routes of CBM administration, EB (patho-)physiologies and symptoms.

Finally, we recognize that there may have been a selection bias as the responses may comprise patients reporting very positive or very negative experiences. The retrospective reporting methodology makes this study vulnerable to a recall bias, and is also cause to interpret the findings with caution. Furthermore, these experiences are not standardized and there is a possibility that participants are benefitting from a placebo effect, underscoring the need for future controlled studies.

## Conclusion

In conclusion, this study highlights the use and perceived multidimensional beneficial effects of treatments with CBMs by EB patients on EB symptoms and disease process. Future prospective controlled clinical studies are warranted to elucidate the potential role of CBMs in EB treatment.

## Supplementary Information

**Additional file 1:Appendix 1:** CBM&EB REDCap Survey.

## Data Availability

The datasets used and/or analysed during the current study are available from the corresponding author on reasonable request.

## Abbreviations

CB1: Cannabinoid receptor 1
CB2: Cannabinoid receptor 2
CBD: Cannabidiol
CBM: Cannabinoid-based medicine
CI: Confidence interval
DDEB: Dominant dystrophic epidermolysis bullosa
DEB: Dystrophic epidermolysis bullosa
EB: Epidermolysis bullosa
EBS: Epidermolysis bullosa simplex
ECS: Endocannabinoid system
IQR: Interquartile range
JEB: Junctional epidermolysis bullosa
NRS: Numeric rating scale
RDEB: Recessive dystrophic epidermolysis bullosa
SCC: Squamous cell carcinoma
SD: Standard deviation
THC: Delta-9-tetrahydrocannabinol
TRP: Transient receptor potential
