# Supplementary material for: Cannabinoid use and effects in patients with epidermolysis bullosa: an international cross-sectional survey study
Source: Orphanet J Rare Dis. 2021 Sep 6;16:377. doi: 10.1186/s13023-021-02010-0 (PMC8419930; doi:10.1186/s13023-021-02010-0)
Supplement: Supplementary file 1 — Additional file 1:Appendix 1: CBM&EB REDCap Survey. [file 13023_2021_2010_MOESM1_ESM.pdf]

Stanford (US) and Groningen (NL) EB Cannabis Survey

Please complete the survey below.

Thank you!

Please select age of EB patient:	◯ Less than 7 years old<br>◯ 7-18 years old<br>◯ Over 18 years old
Please read the following consent form.<br><br>[Attachment: "Cannabis Survey Consent 1-25-2020.pdf"]	
If you agree to participate in this research, please click below.	◯ Yes, I agree to participate in this research study.
Dear Parent or Guardian: Please read the following consent form<br><br>[Attachment: "Cannabis Survey Consent 1-25-2020.pdf"]	
Dear Parent or Guardian: If you agree to allow your child to participate in this research, please click below	◯ Yes, I allow my child to participate in this research study
Dear Patient: Please read the following assent form.<br><br>[Attachment: "Cannabis Survey Assent 1-25-2020.pdf"]	
If you agree to participate in this research, please click below.	◯ Yes, I agree to participate in this research study.

Stanford University (US) and University Medical Center Groningen (NL) EB Cannabis Survey

Throughout the survey, the terms "cannabis" or "cannabis products" will be used to refer to cannabis, marijuana, THC, CBD, and all cannabinoid products.
We will ask questions about dosage, so it may be helpful to gather the packaging for your cannabis product(s) before starting the survey.

Who is completing this survey?	◯ EB patient<br>◯ Parent / guardian / caregiver on behalf of EB patient
Age of EB patient: (select one)	◯ 0-6 years<br>◯ 7-12 years<br>◯ 13-17 years<br>◯ 18-25 years<br>◯ 26-34 years<br>◯ 35 years or older
Sex of EB patient	◯ Male<br>◯ Female<br>◯ Prefer not to answer<br>◯ Other
Please describe your sex	__________________________________

Country:

- ◯ Prefer not to answer
- ◯ United States of America
- ◯ Afghanistan
- ◯ Åland Islands
- ◯ Albania
- ◯ Algeria
- ◯ Andorra
- ◯ Angola
- ◯ Anguilla
- ◯ Antarctica
- ◯ Antigua & Barbuda
- ◯ Argentina
- ◯ Armenia
- ◯ Aruba
- ◯ Ascension Island
- ◯ Australia
- ◯ Austria
- ◯ Azerbaijan
- ◯ Bahamas
- ◯ Bahrain
- ◯ Bangladesh
- ◯ Barbados
- ◯ Belarus
- ◯ Belgium
- ◯ Belize
- ◯ Benin
- ◯ Bermuda
- ◯ Bhutan
- ◯ Bolivia
- ◯ Bosnia & Herzegovina
- ◯ Botswana
- ◯ Brazil
- ◯ British Indian Ocean Territory
- ◯ British Virgin Islands
- ◯ Brunei
- ◯ Bulgaria
- ◯ Burkina Faso
- ◯ Burundi
- ◯ Cambodia
- ◯ Cameroon
- ◯ Canada
- ◯ Canary Islands
- ◯ Cape Verde
- ◯ Caribbean Netherlands
- ◯ Cayman Islands
- ◯ Central African Republic
- ◯ Ceuta & Melilla
- ◯ Chad
- ◯ Chile
- ◯ China
- ◯ Christmas Island
- ◯ Cocos (Keeling) Islands
- ◯ Colombia
- ◯ Comoros
- ◯ Congo - Brazzaville
- ◯ Congo - Kinshasa
- ◯ Cook Islands
- ◯ Costa Rica
- ◯ Côte d'Ivoire
- ◯ Croatia
- ◯ Cuba
- ◯ Curaçao
- ◯ Cyprus
- ◯ Czechia
- ◯ Denmark
- ◯ Diego Garcia
- ◯ Djibouti
- ◯ Dominica
- ◯ Dominican Republic

◯ Ecuador
◯ Egypt
◯ El Salvador
◯ Equatorial Guinea
◯ Eritrea
◯ Estonia
◯ Ethiopia
◯ Falkland Islands
◯ Faroe Islands
◯ Fiji
◯ Finland
◯ France
◯ French Guiana
◯ French Polynesia
◯ French Southern Territories
◯ Gabon
◯ Gambia
◯ Georgia
◯ Germany
◯ Ghana
◯ Gibraltar
◯ Greece
◯ Greenland
◯ Grenada
◯ Guadeloupe
◯ Guam
◯ Guatemala
◯ Guernsey
◯ Guinea
◯ Guinea-Bissau
◯ Guyana
◯ Haiti
◯ Honduras
◯ Hong Kong SAR China
◯ Hungary
◯ Iceland
◯ India
◯ Indonesia
◯ Iran
◯ Iraq
◯ Ireland
◯ Israel
◯ Italy
◯ Jamaica
◯ Japan
◯ Jersey
◯ Jordan
◯ Kazakhstan
◯ Kenya
◯ Kiribati
◯ Kosovo
◯ Kuwait
◯ Kyrgyzstan
◯ Laos
◯ Latvia
◯ Lebanon
◯ Lesotho
◯ Liberia
◯ Libya
◯ Liechtenstein
◯ Lithuania
◯ Luxembourg
◯ Macau SAR China
◯ Macedonia
◯ Madagascar
◯ Malawi
◯ Malaysia
◯ Maldives
◯ Mali
◯ Malta
◯ Marshall Islands

◯ Martinique
◯ Mauritania
◯ Mauritius
◯ Mayotte
◯ Mexico
◯ Micronesia
◯ Moldova
◯ Monaco
◯ Mongolia
◯ Montenegro
◯ Montserrat
◯ Morocco
◯ Mozambique
◯ Myanmar (Burma)
◯ Namibia
◯ Nauru
◯ Nepal
◯ Netherlands
◯ New Caledonia
◯ New Zealand
◯ Nicaragua
◯ Niger
◯ Nigeria
◯ Niue
◯ Norfolk Island
◯ North Korea
◯ Northern Mariana Islands
◯ Norway
◯ Oman
◯ Pakistan
◯ Palau
◯ Palestinian Territories
◯ Panama
◯ Papua New Guinea
◯ Paraguay
◯ Peru
◯ Philippines
◯ Pitcairn Islands
◯ Poland
◯ Portugal
◯ Puerto Rico
◯ Qatar
◯ Réunion
◯ Romania
◯ Russia
◯ Rwanda
◯ Samoa
◯ San Marino
◯ São Tomé & Príncipe
◯ Saudi Arabia
◯ Senegal
◯ Serbia
◯ Seychelles
◯ Sierra Leone
◯ Singapore
◯ Sint Maarten
◯ Slovakia
◯ Slovenia
◯ Solomon Islands
◯ Somalia
◯ South Africa
◯ South Georgia & South Sandwich Islands
◯ South Korea
◯ South Sudan
◯ Spain
◯ Sri Lanka
◯ St. Barthélemy
◯ St. Helena
◯ St. Kitts & Nevis
◯ St. Lucia
◯ St. Martin

◯ St. Pierre & Miquelon
◯ St. Vincent & Grenadines
◯ Sudan
◯ Suriname
◯ Svalbard & Jan Mayen
◯ Swaziland
◯ Sweden
◯ Switzerland
◯ Syria
◯ Taiwan
◯ Tajikistan
◯ Tanzania
◯ Thailand
◯ Timor-Leste
◯ Togo
◯ Tokelau
◯ Tonga
◯ Trinidad & Tobago
◯ Tristan da Cunha
◯ Tunisia
◯ Turkey
◯ Turkmenistan
◯ Turks & Caicos Islands
◯ Tuvalu
◯ Uganda
◯ Ukraine
◯ United Arab Emirates
◯ United Kingdom
◯ Uruguay
◯ Uzbekistan
◯ Vanuatu
◯ Vatican City
◯ Venezuela
◯ Vietnam
◯ Wallis & Futuna
◯ Western Sahara
◯ Yemen
◯ Zambia
◯ Zimbabwe

State

- ◯ Prefer not to answer
- ◯ Alabama
- ◯ Alaska
- ◯ Arizona
- ◯ Arkansas
- ◯ California
- ◯ Colorado
- ◯ Connecticut
- ◯ Delaware
- ◯ Florida
- ◯ Georgia
- ◯ Hawaii
- ◯ Idaho
- ◯ Illinois
- ◯ Indiana
- ◯ Iowa
- ◯ Kansas
- ◯ Kentucky
- ◯ Louisiana
- ◯ Maine
- ◯ Maryland
- ◯ Massachusetts
- ◯ Michigan
- ◯ Minnesota
- ◯ Mississippi
- ◯ Missouri
- ◯ Montana
- ◯ Nebraska
- ◯ Nevada
- ◯ New Hampshire
- ◯ New Jersey
- ◯ New Mexico
- ◯ New York
- ◯ North Carolina
- ◯ North Dakota
- ◯ Ohio
- ◯ Oklahoma
- ◯ Oregon
- ◯ Pennsylvania
- ◯ Rhode Island
- ◯ South Carolina
- ◯ South Dakota
- ◯ Tennessee
- ◯ Texas
- ◯ Utah
- ◯ Vermont
- ◯ Virginia
- ◯ Washington
- ◯ West Virginia
- ◯ Wisconsin
- ◯ Wyoming
- ◯ American Samoa
- ◯ District of Columbia
- ◯ Federated States of Micronesia
- ◯ Guam
- ◯ Marshall Islands
- ◯ Northern Mariana Islands
- ◯ Palau
- ◯ Puerto Rico
- ◯ Virgin Islands

Is cannabis legal where you live?	◯ It is legal to use medically only (prescribed by a doctor)<br>◯ It is legal to use recreationally (I can buy it at a shop / dispensary)<br>◯ It is legal to use recreationally and medically<br>◯ It is not legal<br>◯ I don't know<br>◯ Prefer not to answer<br>◯ Other
Please describe the legality of cannabis where you live	__________________________________
EB Subtype	◯ Recessive Dystrophic EB<br>◯ Dominant Dystrophic EB<br>◯ Junctional EB<br>◯ EB Simplex<br>◯ Kindler Syndrome<br>◯ I don't know<br>◯ Other
Please describe your type of EB	__________________________________
How severe do you consider your EB?	◯ Very Mild<br>◯ Mild<br>◯ Moderate<br>◯ Severe<br>◯ Very Severe

Cannabis Use

Do you currently use cannabis for your EB?	◯ Yes<br>◯ No
Have you ever used cannabis for your EB?	◯ Yes<br>◯ No
Do you have any questions or recommendations for the scientific community? Please write them in the text box.	__________________________________
Where do you get your information about medical cannabis?	__________________________________
Do you have any other comments about cannabis and EB that you would like to share? If so, please write them in the text box.	__________________________________
Click here to end the survey	◯ Survey end
When did you stop using cannabis?	◯ Less than 6 months ago<br>◯ 6 months - 1 year ago<br>◯ 1 - 5 years ago<br>◯ Over 5 years ago
Why did you stop using cannabis? (select all that apply)	☐ It was too expensive<br>☐ Because it was illegal<br>☐ I could not use it in the workplace / at school<br>☐ Some people around me did not like that I was using it<br>☐ I did not like the (side) effects of using it<br>☐ It did not help my EB<br>☐ I found a better medication<br>☐ I do not know<br>☐ Other
Please describe why you stopped using cannabis	___________________________
Why did you decide to use cannabis?	__________________________________

Type of Cannabis Products

What type(s) of cannabis products have you used? (select all that apply)

- ☐ Flower (e.g. marijuana, leaf, bud, weed)
- ☐ Oil or paste (e.g. vape pen cartridge, topical oil or cream)
- ☐ Edible (e.g. food, cookie, brownie)
- ☐ Pill (e.g. tablet, capsule)
- ☐ Drink (e.g. tea)
- ☐ Tincture
- ☐ Suppository
- ☐ Other / not certain

Please describe the type of cannabis product(s) you have used

____

By what route of administration have you used cannabis? (select all that apply)

- ☐ Topical (applied to the skin). Examples: oil, cream, spray, lotion, foam, mixture
- ☐ Inhaled. Examples: smoked (in a joint, through a pipe/bong), vaporized (e-cigarette, vape pen, volcano)
- ☐ Ingested / swallowed. Examples: edibles (food, brownie, cake), drink (tea), medicine taken by mouth (pill)
- ☐ Sublingual (placed under the tongue). Examples: tincture, oil, spray
- ☐ Suppository (rectally, vaginally)
- ☐ Other

What type of topical cannabis do you use? (select all that apply)

- ☐ Oil
- ☐ Cream
- ☐ Spray
- ☐ Lotion
- ☐ Foam
- ☐ Mixture
- ☐ Other

Please describe what is in the mixture:

____

Please describe the type of topical cannabis you use.

____

Where do you apply topical cannabis? (select all that apply)

- ☐ To wounds
- ☐ To unwounded skin
- ☐ To painful thickened skin (keratoderma)
- ☐ Other

Please describe where you apply topical cannabis

____

What information do you have about the contents of the topical cannabis product? (please include all information that you know)

- ☐ % THC
- ☐ % CBD
- ☐ Concentration
- ☐ Milligrams THC
- ☐ Milligrams CBD
- ☐ Ratio of THC to CBD
- ☐ Ratio of CBD to THC
- ☐ Other / Don't know

What is the % THC in the topical product?	__________________
What is the % CBD in the topical product?	__________________
What is the concentration of the topical product?	__________________
How many milligrams of THC are in the topical product?	__________________
How many milligrams of CBD are in the topical product?	__________________
What is the ratio of THC to CBD in the topical product?	__________________
What is the ratio of CBD to THC in the topical product?	__________________
Please describe any additional information you know about the THC or CBD contents of the topical product:	__________________
What is the brand name of the topical product?	__________________
What is the volume (size) of the container (e.g. tube / jar) of the topical cannabis product?	__________________
Select units for the container volume:	◯ milliliter (mL)<br>◯ liter (L)<br>◯ other
Describe "other" unit:	__________________
How long does it take you to go through one container (e.g. tube / jar)?	__________________
Please upload a photograph of the front label of the topical product (if able)	
Please upload a photograph of the back label of the topical product (if able)	
What methods do you use to inhale cannabis? (select all that apply)	☐ Smoked (e.g. in a joint, through a pipe / bong)<br>☐ Vaporized (e.g. e-cigarette, vape pen, volcano)<br>☐ Other
Please describe the method to inhale cannabis	__________________

How much do you inhale in a single session, or each time you use it?
(if you do not know, type "0") (indicate units in the next question)

____

What are the units for how much cannabis you inhale at a single time?

- ◯ millligrams
- ◯ grams
- ◯ ounces
- ◯ I don't know
- ◯ other

Please describe the units for how much cannabis you inhale at a single time

____

What information do you have about the contents of the cannabis product that you inhale? (please include all information that you know)

- ☐ % THC
- ☐ % CBD
- ☐ Concentration
- ☐ Milligrams THC
- ☐ Milligrams CBD
- ☐ Ratio of THC to CBD
- ☐ Ratio of CBD to THC
- ☐ Other / Don't know

What is the % THC in the product you inhale?

____

What is the % CBD in the product you inhale?

____

What is the concentration of the product you inhale?

____

How many milligrams of THC are in the product you inhale?

____

How many milligrams of CBD are in the product you inhale?

____

What is the ratio of THC to CBD in the product you inhale?

____

What is the ratio of CBD to THC in the product you inhale?

____

Please describe any additional information you know about the THC or CBD contents of the product you inhale

____

What is the brand name of the product you inhale?

____

Please upload a photograph of the front label of the inhaled product (if able)

Please upload a photograph of the back label of the inhaled product (if able)

What types of cannabis do you ingest? (select all that apply)	☐ Edibles (e.g. food, brownie)<br>☐ Drink (e.g. tea)<br>☐ Medicine taken by mouth (e.g. pill)<br>☐ Other
Please describe the method to ingest cannabis	__________________________
How much do you ingest at a single time? (indicate units in the next question)	__________________________
What are the units for how much cannabis you ingest at a single time?	◯ droplets<br>◯ milliliters<br>◯ milligrams<br>◯ grams<br>◯ ounces<br>◯ other
Please describe the units for how much cannabis you ingest at a single time	__________________________
What information do you have about the contents of the cannabis product that you ingest? (please include all information that you know)	☐ % THC<br>☐ % CBD<br>☐ Concentration<br>☐ Milligrams THC<br>☐ Milligrams CBD<br>☐ Ratio of THC to CBD<br>☐ Ratio of CBD to THC<br>☐ Other / Don't know
What is the % THC in the ingested product?	__________________________
What is the % CBD in the ingested product?	__________________________
What is the concentration of the ingested product?	__________________________
How many milligrams of THC are in the ingested product?	__________________________
How many milligrams of CBD are in the ingested product?	__________________________
What is the ratio of THC to CBD in the ingested product?	__________________________
What is the ratio of CBD to THC in the ingested product?	__________________________
Please describe any additional information you know about the THC or CBD contents of the ingested product	__________________________
What is the brand name of the product you ingest?	__________________________

Please upload a photograph of the front label of the ingested product (if able)	
Please upload a photograph of the back label of the ingested product (if able)	
What type of sublingual cannabis do you use? (select all that apply)	☐ Tincture<br>☐ Spray<br>☐ Oil<br>☐ Other
Please describe the type of cannabis that you use sublingually	__________________
How much do you use sublingually at a single time? (indicate units in the next question)	__________________
What are the units for how much cannabis you use sublingually at a single time?	○ droplets<br>○ milliliters<br>○ milligrams<br>○ grams<br>○ ounces<br>○ other
Please describe the units for how much cannabis you use sublingually at a single time?	__________________
What information do you have about the contents of the cannabis product that you use sublingually? (please include all information that you know)	☐ % THC<br>☐ % CBD<br>☐ Concentration<br>☐ Milligrams THC<br>☐ Milligrams CBD<br>☐ Ratio of THC to CBD<br>☐ Ratio of CBD to THC<br>☐ Other / Don't know
What is the % THC in the sublingual product?	__________________
What is the % CBD in the sublingual product?	__________________
What is the concentration of the sublingual product?	__________________
How many milligrams of THC are in the sublingual product?	__________________
How many milligrams of CBD are in the sublingual product?	__________________
What is the ratio of THC to CBD in the sublingual product?	__________________
What is the ratio of CBD to THC in the sublingual product?	__________________

Please describe any additional information you know about the THC or CBD contents of the sublingual product.	________________________
What is the brand name of the sublingual product?	________________________
Please upload a photograph of the front label of the sublingual product.	
Please upload a photograph of the back label of the sublingual product.	
Where do you use the suppository? (check all that apply)	☐ Rectally<br>☐ Vaginally<br>☐ Other
Please describe where you use the suppository	________________________
How much do you use as a suppository at a single time? (indicate units in the next question)	________________________
What are the units for how much cannabis you use as a suppository at a single time?	◯ Droplets<br>◯ Milliliters<br>◯ Milligrams<br>◯ Grams<br>◯ Ounces<br>◯ Other
Please describe the units for how much cannabis you use as a suppository at a single time	________________________
What information do you have about the contents of the cannabis product that you use as a suppository? (please include all information that you know)	☐ % THC<br>☐ % CBD<br>☐ Concentration<br>☐ Milligrams THC<br>☐ Milligrams CBD<br>☐ Ratio of THC to CBD<br>☐ Ratio of CBD to THC<br>☐ Other / Don't know
What is the % THC in the suppository product?	________________________
What is the % CBD in the suppository product?	________________________
What is the concentration of the suppository product?	________________________
How many milligrams of THC are in the suppository product?	________________________

How many milligrams of CBD are in the suppository product? __________________________________

What is the ratio of THC to CBD in the suppository product? __________________________________

What is the ratio of CBD to THC in the suppository product? __________________________________

Please describe any additional information you know about the THC or CBD contents of the suppository product? __________________________________

What is the brand name of the suppository product? __________________________________

Please upload a photograph of the front label of the suppository product (if able)

Please upload a photograph of the back label of the suppository product (if able)

Please describe the "other" cannabis product that you use __________________________________

How much do you use at a single time of the "other" cannabis product? __________________________________

What information do you have about the contents of the "other" cannabis product? (please include all information you know)

- ☐ % THC
- ☐ % CBD
- ☐ Concentration
- ☐ Milligrams THC
- ☐ Milligrams CBD
- ☐ Ratio of THC to CBD
- ☐ Ratio of CBD to THC
- ☐ Other / Don't know

What is the % THC in the "other" product? __________________________________

What is the % CBD in the "other" product? __________________________________

What is the concentration of the "other" product? __________________________________

How many milligrams of THC are in the "other" product? __________________________________

How many milligrams of CBD are in the "other" product? __________________________________

What is the ratio of THC to CBD in the "other" product? __________________________________

What is the ratio of CBD to THC in the "other" product?	__________________
Please describe any additional information you know about the THC or CBD contents of the "other" product.	__________________
What is the brand name of the "other" product?	__________________
Please upload a photograph of the front label of the "other" product.	
Please upload a photograph of the back label of the "other" product.	
Of your methods of administration, which do you prefer?	◯ Topical (i.e. applied to the skin)<br>◯ Inhaled (i.e. smoked)<br>◯ Ingested (i.e. food / drink, pill)<br>◯ Sublingual (i.e. under the tongue)<br>◯ Suppository (i.e. rectal, vaginal)<br>◯ Other
Please specify the route that you prefer	__________________
Please include any comments on the contents of the product(s) or the route of administration that you prefer:	______________________

Frequency / Duration of Use

How long have you used cannabis? Please estimate if you are not sure.	◯ Less than 6 months<br>◯ 6 months to 1 year<br>◯ 1 to 5 years<br>◯ Greater than 5 years
How often do you use cannabis?	◯ Less than once per week<br>◯ Once per week<br>◯ Several times per week<br>◯ Once per day<br>◯ Several times per day<br>◯ Other
Please describe how often you use cannabis	____________________________
Approximately how old were you when you first started using cannabis for medicinal purposes?	____________________________<br>(years)
Did the amount of cannabis that you use (e.g. how much you use at a single time) change over time?	◯ The amount decreased<br>◯ The amount stayed the same<br>◯ The amount increased<br>◯ The amount fluctuated (sometimes increased, sometimes decreased)<br>◯ I don't know
Did your frequency of cannabis use (e.g. how often you use it) change over time?	◯ I used it less frequently<br>◯ It stayed the same<br>◯ I used it more frequently<br>◯ The frequency fluctuated (sometimes more, sometimes less)<br>◯ I don't know

Procurement

Where do you buy your cannabis product(s)? Select all that apply.

☐ Dispensary (e.g. at a cannabis store)
☐ Pharmacy (drug store)
☐ From a friend
☐ Through a social connection
☐ I grow it myself
☐ I get it from the internet
☐ I prefer not to answer
☐ Other

Please describe where you buy your cannabis product(s)

____

How much do you spend per month on cannabis products? (Select currency in the next question)

◯ 0-50
◯ 50-100
◯ 100-200
◯ 200-500
◯ 500-1000
◯ 1000-2000
◯ 2000-5000
◯ More than 5000
◯ I don't know

Select your currency

- ◯ United States dollar
- ◯ British pound
- ◯ Euro
- ◯ Abkhazian apsar
- ◯ Afghan afghani
- ◯ Albanian lek
- ◯ Alderney pound
- ◯ Algerian dinar
- ◯ Angolan kwanza
- ◯ Argentine peso
- ◯ Armenian dram
- ◯ Artsakh dram
- ◯ Aruban florin
- ◯ Ascension pound
- ◯ Australian dollar
- ◯ Azerbaijani manat
- ◯ Bahamian dollar
- ◯ Bahraini dinar
- ◯ Bangladeshi taka
- ◯ Barbadian dollar
- ◯ Belarusian ruble
- ◯ Belize dollar
- ◯ Bermudian dollar
- ◯ Bhutanese ngultrum
- ◯ Bolivian boliviano
- ◯ Bosnia and Herzegovina convertible mark
- ◯ Botswana pula
- ◯ Brazilian real
- ◯ British Virgin Islands dollar
- ◯ Brunei dollar
- ◯ Bulgarian lev
- ◯ Burmese kyat
- ◯ Burundian franc
- ◯ Cambodian riel
- ◯ Canadian dollar
- ◯ Cape Verdean escudo
- ◯ Cayman Islands dollar
- ◯ Central African CFA franc
- ◯ CFP franc
- ◯ Chilean peso
- ◯ Chinese yuan
- ◯ Colombian peso
- ◯ Comorian franc
- ◯ Congolese franc
- ◯ Cook Islands dollar
- ◯ Costa Rican colón
- ◯ Croatian kuna
- ◯ Cuban convertible peso
- ◯ Cuban peso
- ◯ Czech koruna
- ◯ Danish krone
- ◯ Djiboutian franc
- ◯ Dominican peso
- ◯ Eastern Caribbean dollar
- ◯ Egyptian pound
- ◯ Eritrean nakfa
- ◯ Ethiopian birr
- ◯ Falkland Islands pound
- ◯ Faroese króna
- ◯ Fijian dollar
- ◯ Gambian dalasi
- ◯ Georgian lari
- ◯ Ghanaian cedi
- ◯ Gibraltar pound
- ◯ Guatemalan quetzal
- ◯ Guernsey pound
- ◯ Guinean franc
- ◯ Guyanese dollar
- ◯ Haitian gourde

- ◯ Honduran lempira
- ◯ Hong Kong dollar
- ◯ Hungarian forint
- ◯ Icelandic króna
- ◯ Indian rupee
- ◯ Indonesian rupiah
- ◯ Iranian rial
- ◯ Iraqi dinar
- ◯ Israeli new shekel
- ◯ Jamaican dollar
- ◯ Japanese yen
- ◯ Jersey pound
- ◯ Jordanian dinar
- ◯ Kazakhstani tenge
- ◯ Kenyan shilling
- ◯ Kiribati dollar
- ◯ Kuwaiti dinar
- ◯ Kyrgyzstani som
- ◯ Lao kip
- ◯ Lebanese pound
- ◯ Lesotho loti
- ◯ Liberian dollar
- ◯ Libyan dinar
- ◯ Macanese pataca
- ◯ Macedonian denar
- ◯ Malagasy ariary
- ◯ Malawian kwacha
- ◯ Malaysian ringgit
- ◯ Maldivian rufiyaa
- ◯ Manx pound
- ◯ Mauritanian ouguiya
- ◯ Mauritian rupee
- ◯ Mexican peso
- ◯ Micronesian dollar
- ◯ Moldovan leu
- ◯ Mongolian tögrög
- ◯ Moroccan dirham
- ◯ Mozambican metical
- ◯ Namibian dollar
- ◯ Nauruan dollar
- ◯ Nepalese rupee
- ◯ Netherlands Antillean guilder
- ◯ New Taiwan dollar
- ◯ New Zealand dollar
- ◯ Nicaraguan córdoba
- ◯ Nigerian naira
- ◯ Niue dollar
- ◯ North Korean won
- ◯ Norwegian krone
- ◯ Omani rial
- ◯ Pakistani rupee
- ◯ Palauan dollar
- ◯ Panamanian balboa
- ◯ Papua New Guinean kina
- ◯ Paraguayan guaraní
- ◯ Peruvian sol
- ◯ Philippine piso
- ◯ Pitcairn Islands dollar
- ◯ Polish złoty
- ◯ Qatari riyal
- ◯ Romanian leu
- ◯ Russian ruble
- ◯ Rwandan franc
- ◯ Sahrawi peseta
- ◯ Saint Helena pound
- ◯ Samoan tālā
- ◯ Saudi riyal
- ◯ Serbian dinar
- ◯ Seychellois rupee
- ◯ Sierra Leonean leone
- ◯ Singapore dollar

	◯ Solomon Islands dollar<br>◯ Somali shilling<br>◯ Somaliland shilling<br>◯ South African rand<br>◯ South Georgia and the South Sandwich Islands pound<br>◯ South Korean won<br>◯ South Sudanese pound<br>◯ Sri Lankan rupee<br>◯ Sudanese pound<br>◯ Surinamese dollar<br>◯ Swazi lilangeni<br>◯ Swedish krona<br>◯ Swiss franc<br>◯ Syrian pound<br>◯ São Tomé and Príncipe dobra<br>◯ Tajikistani somoni<br>◯ Tanzanian shilling<br>◯ Thai baht<br>◯ Tongan paʻanga<br>◯ Transnistrian ruble<br>◯ Trinidad and Tobago dollar<br>◯ Tristan da Cunha pound<br>◯ Tunisian dinar<br>◯ Turkish lira<br>◯ Turkmenistan manat<br>◯ Tuvaluan dollar<br>◯ Ugandan shilling<br>◯ Ukrainian hryvnia<br>◯ United Arab Emirates dirham<br>◯ Uruguayan peso<br>◯ Uzbekistani soʻm<br>◯ Vanuatu vatu<br>◯ Venezuelan bolívar<br>◯ Vietnamese đồng<br>◯ West African CFA franc<br>◯ Yemeni rial<br>◯ Zambian kwacha
Is your cannabis prescribed by your doctor?	◯ Yes<br>◯ No
Does your doctor know that you use cannabis?	◯ Yes<br>◯ No

Effects of Cannabis

Please refer to the image below when rating your pain.

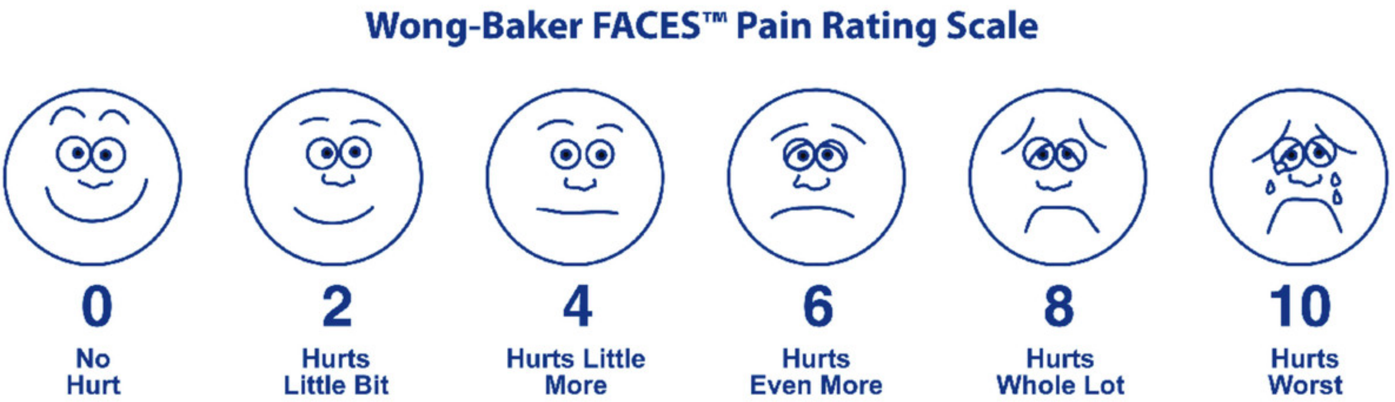

23 Please rate your overall average daily pain from EB prior to cannabis use:

- ◯ 0 (no pain)
- ◯ 1
- ◯ 2
- ◯ 3
- ◯ 4
- ◯ 5
- ◯ 6
- ◯ 7
- ◯ 8
- ◯ 9
- ◯ 10 (worst possible)

24 Please rate your overall average daily pain from EB after using cannabis:

- ◯ 0 (no pain)
- ◯ 1
- ◯ 2
- ◯ 3
- ◯ 4
- ◯ 5
- ◯ 6
- ◯ 7
- ◯ 8
- ◯ 9
- ◯ 10 (worst possible)

Please rate your overall average daily itch from EB prior to using cannabis:

- ◯ 0 (no itch)
- ◯ 1
- ◯ 2
- ◯ 3
- ◯ 4
- ◯ 5
- ◯ 6
- ◯ 7
- ◯ 8
- ◯ 9
- ◯ 10 (worst possible)

Please rate your overall average daily itch from EB after using cannabis:	◯ 0 (no itch)<br>◯ 1<br>◯ 2<br>◯ 3<br>◯ 4<br>◯ 5<br>◯ 6<br>◯ 7<br>◯ 8<br>◯ 9<br>◯ 10 (worst possible)
In general, how has cannabis affected your EB symptoms?	◯ Gives me great relief<br>◯ Gives me a little relief<br>◯ Makes no difference<br>◯ I feel a little worse<br>◯ I feel a lot worse

Effects on Pain

What effect has cannabis had on overall pain?

- ◯ Much improved
- ◯ A little improved
- ◯ No change
- ◯ A little worse
- ◯ A lot worse
- ◯ I don't have pain

What effect has cannabis had on pain from blisters or wounds?

- ◯ Much improved
- ◯ A little improved
- ◯ No change
- ◯ A little worse
- ◯ A lot worse
- ◯ I don't have pain at wounds

What effect has cannabis had on pain from thickened skin (keratoderma)?

- ◯ Much improved
- ◯ A little improved
- ◯ No change
- ◯ A little worse
- ◯ A lot worse
- ◯ I don't have pain from thickened skin / I don't have thickened skin

What effect has cannabis had on pain that is itchy?

- ◯ Much improved
- ◯ A little improved
- ◯ No change
- ◯ A little worse
- ◯ A lot worse
- ◯ I don't have pain that is itchy

What effect has cannabis had on burning pain?

- ◯ Much improved
- ◯ A little improved
- ◯ No change
- ◯ A little worse
- ◯ A lot worse
- ◯ I don't have burning pain

What effect has cannabis had on shooting pain?

- ◯ Much improved
- ◯ A little improved
- ◯ No change
- ◯ A little worse
- ◯ A lot worse
- ◯ I don't have shooting pain

What effect has cannabis had on stabbing pain?

- ◯ Much improved
- ◯ A little improved
- ◯ No change
- ◯ A little worse
- ◯ A lot worse
- ◯ I don't have stabbing pain

What effect has cannabis had on pain during dressing changes?

- ◯ Much improved
- ◯ A little improved
- ◯ No change
- ◯ A little worse
- ◯ A lot worse
- ◯ I don't have pain during dressing changes / I don't do dressing changes

What effect has cannabis had on pain caused by movement (e.g. walking)?	◯ Much improved<br>◯ A little improved<br>◯ No change<br>◯ A little worse<br>◯ A lot worse<br>◯ I don't have pain caused by movement
What effect has cannabis had on background pain (e.g. pain that you have all the time)?	◯ Much improved<br>◯ A little improved<br>◯ No change<br>◯ A little worse<br>◯ A lot worse<br>◯ I don't have background pain
What effect has cannabis had on eye pain?	◯ Much improved<br>◯ A little improved<br>◯ No change<br>◯ A little worse<br>◯ A lot worse<br>◯ I don't have eye pain
What effect has cannabis had on mouth / throat pain?	◯ Much improved<br>◯ A little improved<br>◯ No change<br>◯ A little worse<br>◯ A lot worse<br>◯ I don't have mouth / throat pain
What effect has cannabis had on pain when defecating/pooping?	◯ Much improved<br>◯ A little improved<br>◯ No change<br>◯ A little worse<br>◯ A lot worse<br>◯ I don't have pain when defecating/pooping
What effect has cannabis had on stomach pain?	◯ Much improved<br>◯ A little improved<br>◯ No change<br>◯ A little worse<br>◯ A lot worse<br>◯ I don't have stomach pain
What effect has cannabis had on pain due to skin cancer?	◯ Much improved<br>◯ A little improved<br>◯ No change<br>◯ A little worse<br>◯ A lot worse<br>◯ I don't have pain due to skin cancer / I don't have skin cancer

Effects on Skin

What effect has cannabis had on itchiness?	◯ Much improved<br>◯ A little improved<br>◯ No change<br>◯ A little worse<br>◯ A lot worse<br>◯ I don't have a problem with itching
What effect has cannabis had on wound healing time?	◯ Much improved<br>◯ A little improved<br>◯ No change<br>◯ A little worse<br>◯ A lot worse<br>◯ I don't have wounds
What effect has cannabis had on ease of blistering?	◯ Much improved<br>◯ A little improved<br>◯ No change<br>◯ A little worse<br>◯ A lot worse<br>◯ I don't blister easily
What effect has cannabis had on skin inflammation?	◯ Much improved<br>◯ A little improved<br>◯ No change<br>◯ A little worse<br>◯ A lot worse<br>◯ I don't have problems with skin inflammation
What effect has cannabis had on wound infections?	◯ Much improved<br>◯ A little improved<br>◯ No change<br>◯ A little worse<br>◯ A lot worse<br>◯ I don't get wound infections
What effect has cannabis had on growth of skin cancer?	◯ Much improved<br>◯ A little improved<br>◯ No change<br>◯ A little worse<br>◯ A lot worse<br>◯ I have not had skin cancer

Non-skin effects

What effect has cannabis had on appetite?	◯ Much improved<br>◯ A little improved<br>◯ No change<br>◯ A little worse<br>◯ A lot worse<br>◯ I don't have problems with my appetite
What effect has cannabis had on constipation?	◯ Much improved<br>◯ A little improved<br>◯ No change<br>◯ A little worse<br>◯ A lot worse<br>◯ I don't have constipation
What effect has cannabis had on your ability to move around (e.g. walk)?	◯ Much improved<br>◯ A little improved<br>◯ No change<br>◯ A little worse<br>◯ A lot worse<br>◯ I don't have problems moving around
What effect has cannabis had on your overall mood?	◯ Much improved<br>◯ A little improved<br>◯ No change<br>◯ A little worse<br>◯ A lot worse<br>◯ I don't have problems with my mood
What effect has cannabis had on anxiety?	◯ Much improved<br>◯ A little improved<br>◯ No change<br>◯ A little worse<br>◯ A lot worse<br>◯ I don't have problems with anxiety
What effect has cannabis had on your ability to relax?	◯ Much improved<br>◯ A little improved<br>◯ No change<br>◯ A little worse<br>◯ A lot worse<br>◯ I don't have problems relaxing
What effect has cannabis had on sleep?	◯ Much improved<br>◯ A little improved<br>◯ No change<br>◯ A little worse<br>◯ A lot worse<br>◯ I don't have problems with sleeping
What effect has cannabis had on energy levels?	◯ Much improved<br>◯ A little improved<br>◯ No change<br>◯ A little worse<br>◯ A lot worse<br>◯ I don't have problems with my energy levels

57 Please describe any other effects cannabis has had on your well being

____

Medication-use:

Did you take any of the following medicines around the same time that you used cannabis?

	no	yes
Over the counter pain medicines. Examples: acetaminophen (Tylenol), paracetamol, aspirin, ibuprofen	◯	◯
Opioid pain medicines. Examples: hydrocodone (Norco), oxycodone (Oxynorm / Oxycontin), tramadol, morphine, codeine, hydromorphine (Dilaudid)	◯	◯
Medicines that treat burning / shooting (neuropathic) pain. Examples: gabapentin, pregabalin (Lyrica), amitryptiline (Elavil), nortryptiline (Pamelor)	◯	◯
Anti-itch medicines. Examples: hydroxyzine (Atarax), diphenhydramine (Benadryl), loratadine (Claritin), fexofenadine (Allegra), cetirizine (Zyrtec), dimetindeen (Fenistil), promethazine (Phenergan), ondansetron (Zofran)	◯	◯
Anti-depressant medicines. Examples: duloxetine (Cymbalta), venlafaxine (Effexor), fluoxetine (Prozac), buproprion (Wellbutrin), citalopram (Celexa), escitalopram (Lexapro), mirtazapine (Remeron), sertraline (Zoloft)	◯	◯
Benzodiazepines (anti-anxiety medicines). Examples: lorazepam (Ativan), diazepam (Valium), alprazolam (Xanax), clonazepam (Klonopin), midazolam (Versed)	◯	◯

Has your need for over the counter pain medicine changed after you started using cannabis?

- ◯ I have been able to stop using this medicine
- ◯ A lot less needed
- ◯ A little less needed
- ◯ No change
- ◯ A little more needed
- ◯ A lot more needed
- ◯ I don't know

Has your need for opioid pain medicine changed after you started using cannabis?	◯ I have been able to stop using this medicine<br>◯ A lot less needed<br>◯ A little less needed<br>◯ No change<br>◯ A little more needed<br>◯ A lot more needed<br>◯ I don't know
Has your need for medicines that treat burning / shooting pain changed after you started using cannabis?	◯ I have been able to stop using this medicine<br>◯ A lot less needed<br>◯ A little less needed<br>◯ No change<br>◯ A little more needed<br>◯ A lot more needed<br>◯ I don't know
Has your need for anti-itch medicines changed after you started using cannabis?	◯ I have been able to stop using this medicine<br>◯ A lot less needed<br>◯ A little less needed<br>◯ No change<br>◯ A little more needed<br>◯ A lot more needed<br>◯ I don't know
Has your need for anti-depressant medicines changed after you started using cannabis?	◯ I have been able to stop using this medicine<br>◯ A lot less needed<br>◯ A little less needed<br>◯ No change<br>◯ A little more needed<br>◯ A lot more needed<br>◯ I don't know
Has your need for benzodiazepines (anti-anxiety medicines) changed after you started using cannabis?	◯ I have been able to stop using this medicine<br>◯ A lot less needed<br>◯ A little less needed<br>◯ No change<br>◯ A little more needed<br>◯ A lot more needed<br>◯ I don't know

Do you / have you needed the following?

	no	yes
Esophageal Dilations	◯	◯
Gastrostomy tube (G-tube)	◯	◯
Wheelchair or walking assistance device	◯	◯

Has your need for esophageal dilatations changed after you started using cannabis?

- ◯ I have been able to stop getting esophageal dilatations
- ◯ A lot less needed
- ◯ A little less needed
- ◯ No change
- ◯ A little more needed
- ◯ A lot more needed
- ◯ I don't know

Has your need for G tube use changed after you started using cannabis?

- ◯ I have been able to stop using my G tube
- ◯ A lot less needed
- ◯ A little less needed
- ◯ No change
- ◯ A little more needed
- ◯ A lot more needed
- ◯ I don't know

Has your need for wheelchair (or walking assistance device) use changed after you started using cannabis?

- ◯ I have been able to stop using a wheelchair / walking assistance device
- ◯ A lot less needed
- ◯ A little less needed
- ◯ No change
- ◯ A little more needed
- ◯ A lot more needed
- ◯ I don't know

Cannabis side-effects: Did / do you experience these side-effects from (medical) cannabis?

	No	Yes	I don't know
Paranoia	◯	◯	◯
Dizziness / lightheadedness	◯	◯	◯
Fatigue	◯	◯	◯
Problems with coordination	◯	◯	◯
Problems with memory / attention	◯	◯	◯
Dry mouth	◯	◯	◯
Dry / red eyes	◯	◯	◯
Cough / wheezing	◯	◯	◯
Nausea / vomiting	◯	◯	◯
Hallicinations / psychosis	◯	◯	◯
Other side effects	◯	◯	◯

Please describe the other side effects you have experienced from cannabis: ______________________________

Would you recommend cannabis to other EB patients? ◯ Yes ◯ No ◯ I prefer not to answer

Do you have any questions or recommendations for the scientific community?
Please write them down in the text-box ______________________________

Where do you get your information about (medicinal) cannabis? ______________________________

Do you have any other comments about cannabis and EB that you would like to share?
If so, please write them down in the text box ______________________________
